# Downregulation of the Gli Transcription Factors Regulator Kif7 Facilitates Cell Survival and Migration of Choriocarcinoma Cells

**DOI:** 10.1371/journal.pone.0108248

**Published:** 2014-09-29

**Authors:** Joanna Ho, Yanan Du, Oscar Gee-Wan Wong, Michelle K. Y. Siu, Karen K. L. Chan, Annie N. Y. Cheung

**Affiliations:** 1 Department of Pathology, The University of Hong Kong, HKSAR, China; 2 Department of Obstetrics and Gynaecology, The University of Hong Kong, HKSAR, China; 3 Department of Pathology, The University of Hong Kong – Shenzhen Hospital, Shenzhen, China; Hospital Authority, China

## Abstract

The kinesin protein Kif7 has been recognized as an integral component of hedgehog signalling. Aberrant activation of hedgehog signalling has been implicated in many human solid tumours. Gestational trophoblastic disease includes frankly malignant choriocarcinoma and potentially malignant hydatidiform mole. Here we investigated the hedgehog signalling components expression profiles in gestational trophoblastic disease. Downregulation of Gli1, Gli2, Gli3 and Kif7 was demonstrated in clinical samples of choriocarcinoma and hydatidiform moles as well as choriocarcinoma cell lines when compared with normal placentas. Ectopic expression of Kif7 in two choriocarcinoma cell lines JAR and JEG-3 led to a decrease in cell growth and increase in apoptosis demonstrated by MTT and TUNEL assays, respectively. Overexpression of Kif7 also led to suppressed cell migration through transwell assay. In contrast, knocking down Kif7 in HTR-8/SVneo, an immortalized trophoblast cell line, increased cell number over time and increased the migratory ability of the cells. Taken together, Kif7 may contribute to pathogenesis of gestational trophoblastic disease through enhancing survival and promoting dissemination of trophoblasts.

## Introduction

Gestational trophoblastic disease (GTD) is a family of pregnancy-related diseases characterized by abnormal proliferation of placental trophoblasts [1], [2]. There are at least five types of GTD with distinct genetic, histopathological and clinical features: hydatidiform mole (HM), invasive mole (IM), choriocarcinoma (CCA), placental site trophoblastic tumour (PSTT), and epithelioid trophoblastic tumour (ETT). HM is a relatively benign condition whereas the others could be considered frankly malignant tumours [3], [4]. Although most HM can be successfully treated with suction evacuation, a significant proportion (8–30%) of HM will subsequently progress into malignant GTD, most commonly CCA, and require chemotherapy. On the other hand, while more than half of all CCA were developed from HM, CCA may also develop *de novo* after normal pregnancy, spontaneous abortion or ectopic pregnancy [5]. There is no definite predictor for malignant progression of HM currently. Identification of patients at risk hence relies on serial human chorionic gonadotropin (hCG) monitoring after suction evacuation. Understanding the pathogenesis of malignant GTD may reveal novel predictive biomarkers and therapeutic targets.

Several lines of evidence suggested that the pathogenesis of GTD may involve dysregulated stem cell activities [6]. For instance, Oct4, a transcription factor critical for maintaining the pluripotency of embryonic stem cells (ESCs), is downregulated in HM and CCA by promoter hypermethylation [7]. Similarly, we discovered that the methylation status of the promoter of *SOX2*, which encodes a transcription factor critical for self-renewal capacity of ESCs, was increased in GTD. This epigenetic change was associated with downregulation of the corresponding transcript and protein levels [8]. On the other hand, Stat3 and Nanog are overexpressed in GTD and contribute to apoptosis regulation and invasion capability of GTD trophoblasts [9], [10]. These findings prompted us to ask whether other stem cell regulation pathways are dysregulated in GTD.

The Hedgehog (HH) signalling pathway is essential for stem cell maintenance and tissue development [11], [12]. Aberrant activation of HH signalling pathway has been documented in various human malignancies including ovarian and endometrial carcinomas as previously reported by our group [13]–[15]. In mammals, there are three HH ligand proteins: Sonic Hedgehog (Shh), Indian Hedgehog (Ihh) and Desert Hedgehog (Dhh). In the absence of ligands, the transmembrane protein receptor Patched (Ptch) inhibits another membrane protein Smoothened (Smo) from activating downstream signalling [16]. Ligand binding to Ptch ultimately leads to activation of the Gli transcription factor family, which in vertebrate consists of Gli1, Gli2, and Gli3, by post-translational proteolytic processing [17]. Activated Gli translocates into the nucleus and subsequently activates or represses target genes expression [18]. Among the three Gli transcription factors in mammal, Gli1 and Gli2 are the major activating Gli (Gli^act^), whereas proteasome cleaved Gli3 is the major repressor Gli (Gli^rep^) [19]. In human malignancies, aberrant activation of HH pathway could be resulted from sustained increase in endogenous expression of HH ligand (ligand-dependent mechanisms) or mutations of Ptch or Smo in the pathway (ligand-independent mechanisms) [20].

In Drosophila, Costal-2 (Cos2) is a Hedgehog signalling component, relaying signals from Smo to Cubitus interruptus (Ci, the only Gli in Drosophila), Fused (Fu), and microtubule [21]. The kinesin protein Kif7 is the mammalian homolog of Cos2. Genetic studies revealed that the function of Kif7 as a regulator of Gli was conserved in mammal [22]. Homozygous knock-out mice of Kif7 exhibited exencephaly and polydactyly which are phenotypes reminiscent of Gli3 knockout [23], [24], suggesting a predominant suppressor role of Kif7 in HH signalling during development. However, Ptch-Kif7 double mutant exhibited a milder phenotype than Ptch knockout (a HH hyper-activation phenotype), suggesting that Kif7 may also participate in HH pathway activation [24].

In this study, the expression profile of activator/repressor Gli transcription factors and regulator (Gli1, Gli2, Gli3 and Kif7) in GTD was assessed. The functional implications of Kif7 dysregulation on cell proliferation, apoptosis, and cell migration/invasion were also investigated.

## Materials and Methods

### Clinical samples and cell lines

Eighty-seven trophoblast samples, including 19 normal first trimester placentas, 10 term placentas, four choriocarcinomas, 50 hydatidiform moles consisting of 11 partial moles and 39 complete moles were retrieved from the archive of the Department of Pathology, The University of Hong Kong, Queen Mary Hospital. First trimester placenta tissues and molar tissues were obtained by suction evacuation while samples of term placentas were collected after uneventful delivery. GTD cases were diagnosed following the commonly used and acceptable histological morphology criteria as previously described [7]. Three human choriocarcinoma cell lines, JEG-3, JAR, and BeWo, were obtained from American type culture collection (Rockville, MD, USA). A normal trophoblastic cell lines TEV-1 was established by immortalization of villous and extravillous trophoblasts by HPV16 E6/E7 gene transfection [25]. Another immortalized trophoblastic cell line HTR-8/SVneo was contributed by Professor Peeyush K. Lala [26]. All cell lines were cultured in Minimum Essential Medium Eagle (Sigma, St. Louis, MO) supplemented with 10% foetal calf serum and 100 U/ml penicillin and streptomycin.

### Ethics Statement

Ethics approval for the use of tissue samples in this study has been obtained from Institutional Review Board of the University of Hong Kong/Hospital Authority Hong Kong West Cluster (HKU/HA HKW IRB). The tissue samples used in this study were archival patient samples stored after their use for diagnosis has been completed. The need for written informed consent from individual patient was waived by HKU/HA HKW IRB.

### RNA extraction and reverse transcription quantitative PCR

RNA extraction was performed on cell lines or frozen placental tissues using TRIzol (Life Technologies) according to the manufacturer’s instruction. 2.5 µg of total RNA was reverse transcribed using Superscript III Reverse Transcriptase (Life Technologies). Reverse transcription quantitative PCR (RT-qPCR) was performed as previously described [7]. Gene expression levels relative to one of the samples (calibrator) were calculated using the ΔΔC_t_ method using GAPDH as the normalization internal control gene. Sequences of primers used were shown in Table 1.

**Table 1:** Primers used for qPCR in this study.

Target	Sequence (5→3)
Gli1	TCTGCCCCCATTGCCCACT
	TACATAGCCCCCAGCCCCATACCTC
Gli2	GGTGACATGGACAACGTGAG
	TCCAGGGACACACACCTACA
Gli3	AGGCTGCACTAAGCGTTACA
	CTTTCTAGTTTTACGTGCTCC
Kif7	CTCTGTGGTCAGCCTGGAAC
	CCTCTTCCACGATGTCGTCG
GAPDH	TGCACCACCAACTGCTTAGC
	GGCATGGACTGTGGTCATGAG
RFX-3	CCTCCCCAGCGACAATTGAA
	TGGGAAGGCTCACTCCTTCT

### Generation of CCA cell lines with stable overexpression of Kif7

The expression plasmid pEF6/V5-His-Kif7 was generated by subcloning the Kif7 ORF from pCR-BluntII-TOPO-Kif7 (Imagenes) into pEF6/V5-His-TOPO (Life Technologies) using BstXI sites. The CCA cell lines JEG-3 and JAR were transfected with pEF6/V5-His-Kif7 or the control vector pEF6/V5-His using Lipofectamine 2000 (Life Technologies). Pooled stable clones were selected by 10 µg/ml blasticidin S.

### Knockdown of Kif7 using siRNAs

Control and Kif7 siRNAs were purchased from IDT (Singapore) (Cat# HSC.RNAI.N198525.12). The siRNAs were transfected into HTR-8/SVneo using Lipofectamine 2000 (Life Technologies) according to the manufacturer’s instruction.

### Protein Extraction and western blotting

Cells were lysed with RIPA buffer with 2 mM N-ethylmaleimide, 2 mM PMSF, 1 mM sodium orthovanadate, 0.1 µM sodium okadat. Protein concentration was determined by protein assay (Bio-Rad). A total of 20 µg protein was resolved by SDS-PAGE and transferred to polyvinylidene difluoride membrane. Antibodies used for detection included anti-Kif7 (Abcam ab95884) and anti-actin (Sigma A5060).

### MTT assay

Stably transfected cells were seeded in 96-well plates at a density of 1×10^4^ cell/well. MTT solution was added to each well 24 hours after seeding and incubated at 37°C for 4 hours. Precipitate was dissolved by dimethyl sulfoxide solution. Absorbance at 590 nm was measured by a plate reader.

### TUNEL assay

TUNEL cell death detection assay (Roche) was performed according to manufacturer’s instruction. Briefly, cells were plated on round glass cover slips in 24-well plates and the cover slips were collected 48 hours after plating. Cells were fixed with 4% paraformaldehyde and permeablized with 0.1% Triton-X. Cells were then incubated in the TUNEL reaction mixture containing TdT and fluorescein-dUTP. Slides were observed under a fluorescence microscope. Apoptotic cell death of each sample was quantified by apoptotic index (number of apoptotic cells/number of total counted cells ×100%).

### Migration/Invasion assay

JEG-3, JAR, or HTR-8/SVneo were trypsinized and resuspended in serum-free MEM. 2.5×10^5^ cells/well were plated in the upper chambers of gelatin-coated transwells. The transwells were then placed in 24-well plates containing MEM. The inserts were harvested at 24 hours for JAR, 66 hours for JEG-3, and 48 hours for HTR-8/SVneo and stained with crystal violet after non-migrated cells were removed.

### Statistical analysis

Statistical significances of the differences between expression levels determined by RT-qPCR and differences in cell number in MTT assay were tested with two-tailed Student’s T-test.

## Results

### Gli transcription factors and the Gli regulator Kif7 are downregulated in GTD

**Figure 1:**
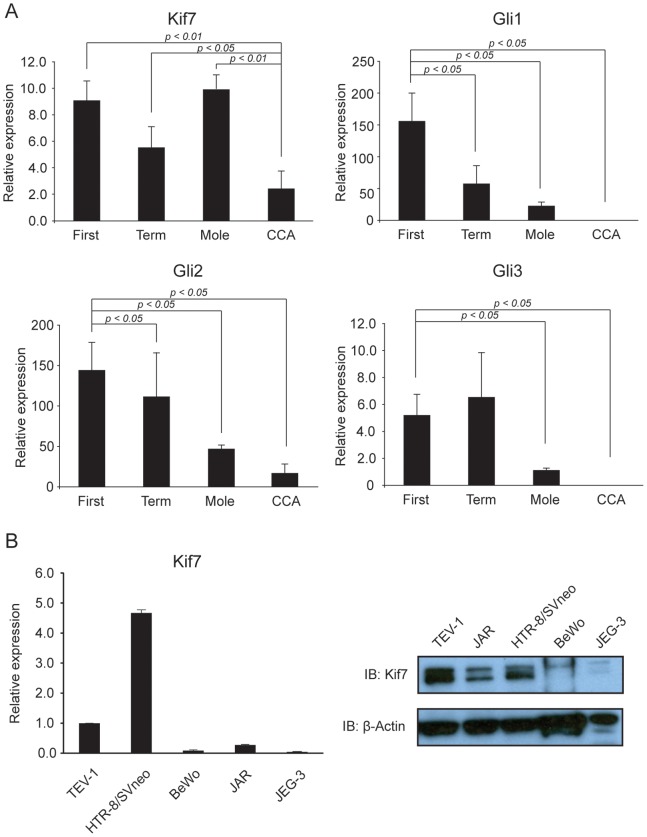
Hedgehog signalling components were differentially regulated in normal and GTD placentas. (A) The levels of Gli transcription factors and Kif7 transcripts in tissue samples of first trimester, term, hydatidiform mole, and choriocarcinoma placentas were compared with RT-qPCR. (B) The level of Kif7 was compared in immortalized trophoblast cell lines (TEV-1, HTR-8/SVneo) and choriocarcinoma cell lines JEG-3, JAR, and BeWo with RT-qPCR (left panel) and western blotting (right panel). Data represent the mean ± SEM of three independent experiments.

The expression levels of Gli1, Gli2, and Gli3 transcripts from first trimester placenta, term placenta, HM, and choriocarcinoma were compared using RT-qPCR. Both Gli1 and Gli2 showed a decreasing trend across the samples in the order of first trimester placenta>term placenta>HM>CCA (Figure 1A). Gli3 level was found to be markedly reduced in HM and CCA when compared with normal placentas although no significant difference between first-trimester and term placenta was detected (Figure 1A).

Unlike the Gli transcription factors, Kif7 showed significant reduction only in CCA samples (Figure 1A). Concurring significant reduction of Kif7 expression was also demonstrated in the three CCA cell lines JEG-3, JAR, and BeWo when compared with normal trophoblast cell lines TEV-1 and HTR-8/SVneo (Figure 1B).

### Kif7 overexpression suppressed cell growth and migration

**Figure 2:**
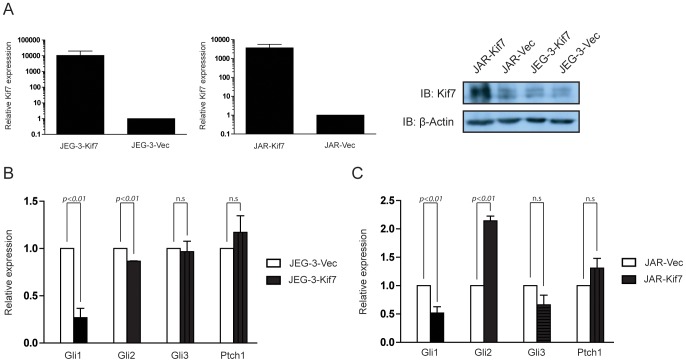
Kif7 overexpression in JEG-3 and JAR dysregulated other HH signalling components. (A) Left panel: RT-qPCR measurement of Kif7 transcript expression in JEG-3 or JAR cells stably transfected with empty vector (JEG-3-Vec, JAR-Vec) or Kif7 expression plasmid (JEG-3-Kif7, JAR-Kif7). Data represent the mean ± SEM of three independent experiments. Right-panel: western blot analysis of Kif7 protein expression in the stably transfected cells. (B) RT-qPCR measurement of Gli1, Gli2, Gli3, and Ptch1 transcripts in the stably transfected cells. Data represent the mean ± SEM of three independent experiments.

JEG-3 and JAR were stably transfected with pEF6/V5-His-Kif7 or the control vector pEF6/V5-His. Efficiency of transfection was evaluated by RT-qPCR which showed that JAR-Kif7 and JEG-3-Kif7 expressed about 1000-fold and 10000-fold more Kif7 transcript respectively when compared with cells stably transfected with the control plasmid (Figure 2A). Kif7 protein expression as analysed by western blotting also showed that JEG-3-Kif7 and JAR-Kif7 expressed much higher level of Kif7 than did JEG-3-Vec and JAR-Vec respectively (Figure 2A).

Next, Gli1, Gli2, Gli3 and Ptch1 transcript levels were quantified by RT-qPCR in CCA cell lines stably transfected with Kif7 to assess HH pathway activity and possible interaction with Kif7. We observed a statistically significant decrease in the expression of Gli1 in both JEG-3 and JAR when Kif7 was overexpressed (Figure 2B and 2C). On the other hand, no obvious change in Gli3 and Ptch1 expression was observed in JEG-3-Kif7 and JAR-Kif7. Gli2 expression was slightly downregulated in JEG-3-Kif7 but markedly upregulated in JAR-Kif7 (Figure 2B and 2C).

**Figure 3:**
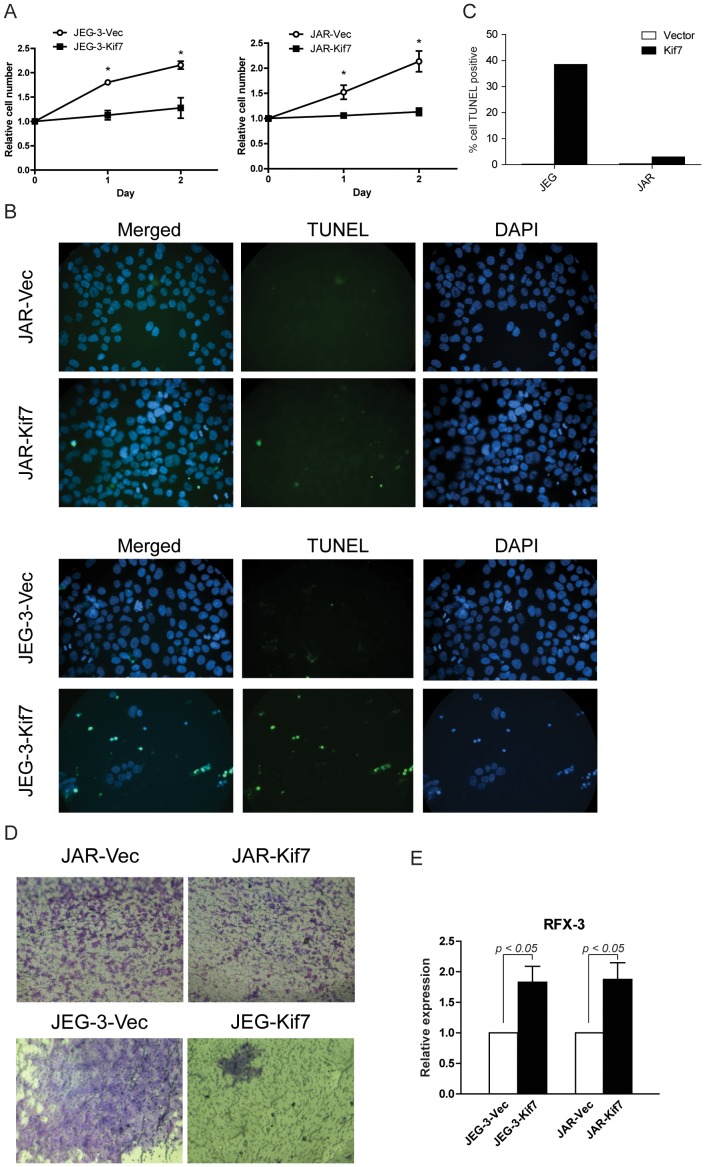
Kif7 overexpression in JEG-3 and JAR led to slower growth rate, increased apoptosis, and reduced mobility. (A) The cell growth of stably transfected cells was measured by MTT assay over three days of culture. Data represented the mean ±95% CI of three independent experiments. * : p<0.01. (B) & (C) Apoptosis in stably transfected cells was detected with TUNEL assay and quantified as percentage of cell being TUNEL positive. (D) Stably transfected JEG-3 or JAR were seeded in the upper chamber of transwells and allowed to migrate through gelatin-coated membrane for 24 hours (JAR) or 66 hours (JEG-3). Migrated cells were stained with crystal violet. (E) The relative expression of RFX-3 was measured with RT-qPCR in stably transfected JEG-3 and JAR. Data represent the mean ± SEM of three independent experiments.

The cell growth of JAR-Kif7 and JEG-3-Kif7 was compared with the control cells using MTT assay over a culturing period of three days. As shown in Figure 3A, JEG-3-Kif7 and JAR-Kif7 grew significantly slower than their control counterparts.

TUNEL assay was performed to assess the apoptotic indices of the stably transfected cells. Both JEG-3 and JAR transfected with the control vector has very low percentage of cell undergoing apoptosis (0.24% and 0.4%, respectively, Figure 3B and 3C). We observed a slight increase in the percentage of cells undergoing apoptosis in JAR-Kif7 (2.98%, Figure 3B and 3C), and a large increase of apoptotic cell in JEG-Kif7 (38.52%, Figure 3B and 3C).

### Kif7 manipulation affected cell migration and invasion

Trophoblasts were known to exhibit varying tendency to invade and metastasize. Therefore we tested the effect of Kif7 overexpression on JEG-3 and JAR cell migration and invasion by transwell migration/invasion assay. Cells migrated through the gelatin coated membranes were fixed and stained with crystal violet 24 hours (for JAR-Vec and JAR-Kif7) or 66 hours (for JEG-3-Vec and JEG-3-Kif7) after seeding. JAR-Vec and JEG-3-Vec readily migrated through membranes (Figure 3D). We observed that JAR-Kif7 and JAR-Vec exhibited similar ability to migrate, whereas Kif7 overexpression drastically inhibited JEG-3 cell migration (Figure 3D).

Mutation in the KIF7 locus has been linked to Joubert syndrome which is a rare, inherited developmental disorder characterized by cerebellar hypoplasia, ataxia, psychomotor delay, and an altered respiratory pattern in the neonatal period. At cellular level, knockdown of Kif7 in polarized retinal epithelial cells led to a dispersed staining pattern of the Golgi apparatus, abnormal centrosome duplications and a reduced number of ciliated cells [27]. Downregulation of Kif7 in CCA may hence affect the ciliogenesis in CCA trophoblasts. We tried to investigate this possibility by monitoring the relative transcript level of a transcriptional regulator of ciliogenesis regulatory factor X 3 (RFX3) in Kif7 stably transfected JEG-3 and JAR. RFX3 is responsible for the transcription of many structural proteins of the primary cilia [28]. Kif7 overexpression in JEG-3 and JAR significantly increased the expression of RFX3 (Figure 3E).

**Figure 4:**
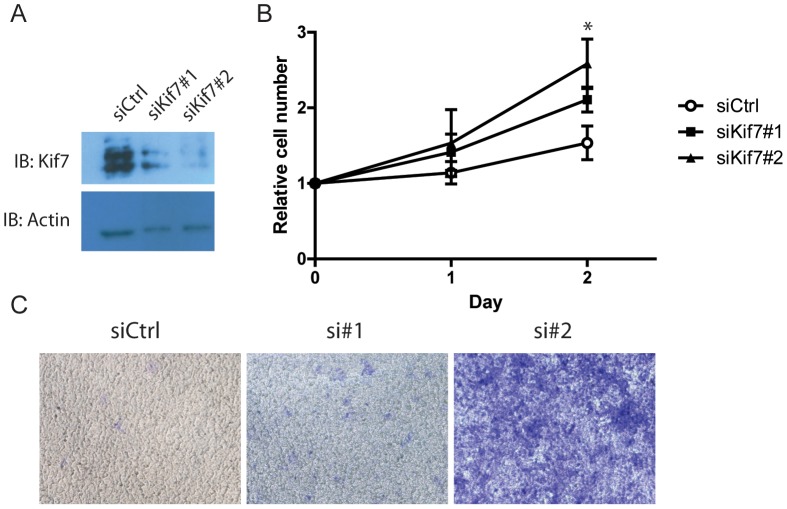
Knockdown of Kif7 in HTR-8/SVneo increased cell growth and cell mobility. (A) HTR-8/SVneo was transiently transfected with control siRNA and two siRNAs against Kif7. The expression of Kif7 in transfected cell was assessed with western blotting. (B) The growth of transfected HTR-8/SVneo was monitored over two days with MTT assay. Data represented the mean ±95% CI of three independent experiments. * : p<0.01. (C) siRNA transfected cells were seeded in the upper chamber of transwells and allowed to migrate through gelatin-coated membrane for 48 hours. Migrated cells were stained with crystal violet.

To confirm the effect of Kif7 on trophoblast cell migration, Kif7 was knockdown in HTR-8/SVneo using siRNA approach. HTR-8/SVneo expressed much higher level of Kif7 compared with JEG-3 and JAR (Figure 1B). Two siRNAs were found to markedly reduce the level of Kif7, with si#2 displayed more potent inhibition of Kif7 expression (Figure 4A). Knocking down Kif7 in HTR-8/SVneo significantly increased the cell growth rate of HTR-8/SVneo (Figure 4B).

HTR-8/SVneo transfected with control siRNA readily migrated through the membrane after 48 hours. Transfection with si#2 drastically increased the number of cell migrated through the membrane, whereas si#1 showed a less obvious effect (Figure 4B).

## Discussion

### HH dysregulation is involved in GTD pathogenesis

Gestational trophoblastic disease, although rare and usually curable, represents an intriguing class of human malignancy. While the cytogenetic mechanisms leading to HM have been fairly well defined, it is unclear how CCA may develop spontaneously from HM or from apparently unremarkable pregnancies. Our previous works have suggested GTD, like many other solid tumours, involves stem cell dysregulation [7]–[10]. The HH signalling pathway has long been known to be important for stem cell self-renewal by contributing to the establishment of stem cell niche [29]. Activation of HH signalling is evident in various human malignancies [30]. However the activity of the pathway in GTD is not known.

The current study described, as far as we know, the first investigation of the role of HH signalling pathway in GTD. We first examined the expression of several components of the HH pathway in clinical samples; then investigated the effect of Kif7, a HH signalling component found dysregulated in clinical samples, in two *invitro* models of CCA, JEG-3 and JAR. Our findings suggest HH signalling is indeed dysregulated in GTD. We focused on the role of Kif7, which exhibited a distinct expression pattern in GTD compared with the Gli transcription factors. Kif7 was found to profoundly inhibit the cell growth and to induce apoptosis of JEG-3 and JAR (Figure 3A–C). Moreover, Kif7 could suppress cell migration and invasion of CCA cells (Figure 3D). These effects were confirmed in knockdown of Kif7 in HTR-8/SVneo, a non-tumorigenic trophoblast model (Figure 4) [26]. These findings suggest Kif7 downregulation to be playing a significant role in trophoblast carcinogenesis.

### Kif7 dysregulation may represent a novel mechanism of HH signalling alteration

Hedgehog signalling pathway is frequently activated in various kinds of malignancies and is often found to contribute to pro-survival mechanisms of cancer cells. HH signalling may be activated either by overexpression of HH ligand, or by disabling mutation of Ptch or activating mutation of Smo [20]. Our discovery that Kif7 is downregulated in CCA may represent a novel mechanism of HH signalling dysregulation in cancer. Although being the mammalian homolog of Drosophila Cos2, the essential upstream signalling component Ci, the involvement of Kif7 in HH signalling was not established until fairly recently [22]–[24]. Kif7 knock-out mice exhibited exencephaly and polydactyly which are phenotypes reminiscent of Gli3 knockout, suggesting that Kif7 predominantly acts as a suppressor of HH signalling during development [23], [24]. Our findings that Kif7 is downregulated imply that HH signalling is activated in CCA.

Kif7 is a member of the kinesin 4 superfamily. It has been found to play important roles in Hedgehog signalling pathway, primary cilium formation, and embryological development. Kif7 mutations or dysregulation was found in diseases such as Joubert syndrome [31]. However, reports on the status or role of Kif7 in human malignancies have been scanty [32]. Our real time PCR experiments demonstrated reduced Kif7 expression in both clinical samples and cell lines of CCA when compared with normal placental trophoblasts or hydatidiform moles (Figure 1). These findings suggested that Kif7 may be important for progression to choriocarcinoma and may serve as a potential genetic marker, especially during surveillance after primary evacuation of molar pregnancy. The usefulness of Kif7 in predicting clinical outcome of GTD needs further investigation on larger number of samples.

### Dysregulation of Gli transcription factors in GTD

Kif7 had been shown to be a critical regulator of the Hedgehog signalling pathway through regulation of Gli transcription factors [23], [24]. In this study, we provided preliminary data suggesting that Kif7 may regulate certain Gli transcription factors at transcript level (Figure 2B and 2C). Gli1 was consistently downregulated by Kif7 overexpression in CCA cell lines. Gli1 is important for the oncogenic effect of HH signalling [33]. In fact, Gli1 was first identified as an amplified, highly expressed factor in human glioma [34], and an oncogenic transcription factor being responsible for the carcinogenesis of sporadic basal cell carcinoma [35]. However, in GTD clinical samples, the expression of Gli1 transcription factors was found to be reduced, and was particularly low in CCA (Figure 1A). This suggests the existence of multiple regulation mechanisms of Gli1 expression in CCA and Kif7 dyregulation is not the dominant mechanism.

The Gli code hypothesis postulates that the three vertebrate Gli transcription factors act together in responding cells to dictate the outcome of HH signalling and other signalling crosstalks [19]. Among the three Gli transcription factors in mammal, Gli1 and Gli2 are the major activating Gli (Gli^act^), whereas proteasome cleaved Gli3 is the major repressor Gli (Gli^rep^) [19]. Interestingly, Gli3 was also strongly downregulated in GTD (Figure 1A), suggesting that it might be a mechanism of elevated HH signaling activity.

## References

[1] Altieri A, Franceschi S, Ferlay J, Smith J, La Vecchia C (2003) Epidemiology and aetiology of gestational trophoblastic diseases. Lancet Oncol 4: 670–678. 14602247 10.1016/s1470-2045(03)01245-2

[2] Cheung AN (2003) Pathology of gestational trophoblastic diseases. Best Pract Res Clin Obstet Gynaecol 17: 849–868. 14614885 10.1016/s1521-6934(03)00094-4

[3] Cheung AN (2009) Gestational Trophoblastic Disease. In: S Robboy, G Mutter, J Prat, R. C Bentley, P Russell and M. C Anderson, editors. Robboy’s Pathology of the Female Reproductive Tract. China: Elsevier Churchill Livingstone. 881–907.

[4] Paradinas FJ, Elston CW (2003) Gestational trophoblastic disease. In: H Fox and M Wells, editors. Haines and Taylor: obstetrical and gynaecological pathology. Edinburgh: Churchill Livingstone. 1359–1430.

[5] Lewis JL Jr (1993) Diagnosis and management of gestational trophoblastic disease. Cancer 71: 1639–1647. 8381709 10.1002/cncr.2820710430

[6] Shih Ie M, Kuo KT (2008) Power of the eternal youth: Nanog expression in the gestational choriocarcinoma. Am J Pathol 173: 911–914. 18755845 10.2353/ajpath.2008.080624 PMC2543060

[7] Zhang HJ, Siu MK, Wong ES, Wong KY, Li AS, et al (2008) Oct4 is epigenetically regulated by methylation in normal placenta and gestational trophoblastic disease. Placenta 29: 549–554. 18440631 10.1016/j.placenta.2008.03.003

[8] Li AS, Siu MK, Zhang H, Wong ES, Chan KY, et al (2008) Hypermethylation of SOX2 gene in hydatidiform mole and choriocarcinoma. Reprod Sci 15: 735–744. 18836133 10.1177/1933719108322433

[9] Chan HY, Siu MK, Zhang HJ, Wong ES, Ngan HY, et al (2008) Activated Stat3 expression in gestational trophoblastic disease: correlation with clinicopathological parameters and apoptotic indices. Histopathology 53: 139–146. 18752497 10.1111/j.1365-2559.2008.03089.x

[10] Siu MK, Wong ES, Chan HY, Ngan HY, Chan KY, et al (2008) Overexpression of NANOG in gestational trophoblastic diseases: effect on apoptosis, cell invasion, and clinical outcome. Am J Pathol 173: 1165–1172. 18772339 10.2353/ajpath.2008.080288 PMC2543083

[11] Ruiz i Altaba A, Sanchez P, Dahmane N (2002) Gli and hedgehog in cancer: tumours, embryos and stem cells. Nat Rev Cancer 2: 361–372. 12044012 10.1038/nrc796

[12] Ingham PW, McMahon AP (2001) Hedgehog signaling in animal development: paradigms and principles. Genes Dev 15: 3059–3087. 11731473 10.1101/gad.938601

[13] Berman DM, Karhadkar SS, Maitra A, Montes De Oca R, Gerstenblith MR, et al (2003) Widespread requirement for Hedgehog ligand stimulation in growth of digestive tract tumours. Nature 425: 846–851. 14520411 10.1038/nature01972

[14] Liao X, Siu MK, Au CW, Chan QK, Chan HY, et al (2009) Aberrant activation of hedgehog signaling pathway contributes to endometrial carcinogenesis through beta-catenin. Mod Pathol 22: 839–847. 19329935 10.1038/modpathol.2009.45

[15] Liao X, Siu MK, Au CW, Wong ES, Chan HY, et al (2009) Aberrant activation of hedgehog signaling pathway in ovarian cancers: effect on prognosis, cell invasion and differentiation. Carcinogenesis 30: 131–140. 19028702 10.1093/carcin/bgn230 PMC7109814

[16] Stone DM, Hynes M, Armanini M, Swanson TA, Gu Q, et al (1996) The tumour-suppressor gene patched encodes a candidate receptor for Sonic hedgehog. Nature 384: 129–134. 8906787 10.1038/384129a0

[17] Briscoe J, Therond PP (2013) The mechanisms of Hedgehog signalling and its roles in development and disease. Nat Rev Mol Cell Biol 14: 416–429. 23719536 10.1038/nrm3598

[18] Jiang J, Hui CC (2008) Hedgehog signaling in development and cancer. Dev Cell 15: 801–812. 19081070 10.1016/j.devcel.2008.11.010 PMC6443374

[19] Ruiz i Altaba A, Mas C, Stecca B (2007) The Gli code: an information nexus regulating cell fate, stemness and cancer. Trends Cell Biol 17: 438–447. 17845852 10.1016/j.tcb.2007.06.007 PMC2601665

[20] Pasca di Magliano M, Hebrok M (2003) Hedgehog signalling in cancer formation and maintenance. Nat Rev Cancer 3: 903–911. 14737121 10.1038/nrc1229

[21] Robbins DJ, Nybakken KE, Kobayashi R, Sisson JC, Bishop JM, et al (1997) Hedgehog elicits signal transduction by means of a large complex containing the kinesin-related protein costal2. Cell 90: 225–234. 9244297 10.1016/s0092-8674(00)80331-1

[22] Cheung HO, Zhang X, Ribeiro A, Mo R, Makino S, et al (2009) The kinesin protein Kif7 is a critical regulator of Gli transcription factors in mammalian hedgehog signaling. Sci Signal 2: ra29. 19549984 10.1126/scisignal.2000405

[23] Endoh-Yamagami S, Evangelista M, Wilson D, Wen X, Theunissen JW, et al (2009) The mammalian Cos2 homolog Kif7 plays an essential role in modulating Hh signal transduction during development. Curr Biol 19: 1320–1326. 19592253 10.1016/j.cub.2009.06.046

[24] Liem KF Jr, He M, Ocbina PJ, Anderson KV (2009) Mouse Kif7/Costal2 is a cilia-associated protein that regulates Sonic hedgehog signaling. Proc Natl Acad Sci U S A 106: 13377–13382. 19666503 10.1073/pnas.0906944106 PMC2726420

[25] Feng HC, Choy MY, Deng W, Wong HL, Lau WM, et al (2005) Establishment and characterization of a human first-trimester extravillous trophoblast cell line (TEV-1). J Soc Gynecol Investig 12: e21–32. 10.1016/j.jsgi.2005.02.008 15866109

[26] Graham CH, Hawley TS, Hawley RG, MacDougall JR, Kerbel RS, et al (1993) Establishment and characterization of first trimester human trophoblast cells with extended lifespan. Exp Cell Res 206: 204–211. 7684692 10.1006/excr.1993.1139

[27] Dafinger C, Liebau MC, Elsayed SM, Hellenbroich Y, Boltshauser E, et al (2011) Mutations in KIF7 link Joubert syndrome with Sonic Hedgehog signaling and microtubule dynamics. J Clin Invest 121: 2662–2667. 21633164 10.1172/JCI43639 PMC3223820

[28] Choksi SP, Lauter G, Swoboda P, Roy S (2014) Switching on cilia: transcriptional networks regulating ciliogenesis. Development 141: 1427–1441. 24644260 10.1242/dev.074666

[29] Yen TH, Wright NA (2006) The gastrointestinal tract stem cell niche. Stem cell reviews 2: 203–212. 17625256 10.1007/s12015-006-0048-1

[30] Taipale J, Beachy PA (2001) The Hedgehog and Wnt signalling pathways in cancer. Nature 411: 349–354. 11357142 10.1038/35077219

[31] Putoux A, Thomas S, Coene KL, Davis EE, Alanay Y, et al (2011) KIF7 mutations cause fetal hydrolethalus and acrocallosal syndromes. Nat Genet 43: 601–606. 21552264 10.1038/ng.826 PMC3674836

[32] Li ZJ, Nieuwenhuis E, Nien W, Zhang X, Zhang J, et al (2012) Kif7 regulates Gli2 through Sufu-dependent and -independent functions during skin development and tumorigenesis. Development 139: 4152–4161. 23034632 10.1242/dev.081190

[33] Kasper M, Regl G, Frischauf AM, Aberger F (2006) GLI transcription factors: mediators of oncogenic Hedgehog signalling. Eur J Cancer 42: 437–445. 16406505 10.1016/j.ejca.2005.08.039

[34] Kinzler KW, Bigner SH, Bigner DD, Trent JM, Law ML, et al (1987) Identification of an amplified, highly expressed gene in a human glioma. Science 236: 70–73. 3563490 10.1126/science.3563490

[35] Dahmane N, Lee J, Robins P, Heller P, Ruiz i Altaba A (1997) Activation of the transcription factor Gli1 and the Sonic hedgehog signalling pathway in skin tumours. Nature 389: 876–881. 9349822 10.1038/39918

